# Complement Activation in BK Polyoma Virus Nephropathy: What’s the Story?

**DOI:** 10.1016/j.ekir.2025.05.019

**Published:** 2025-05-17

**Authors:** M. Barry Stokes

**Affiliations:** 1 Department of Pathology, Columbia University Irving Medical Center, New York, New York, USA

See Clinical Research on Page 2369

Most people are infected with BK polyoma virus (BKPyV) in early childhood (> 75% of adults have immunity) resulting in lifelong latent urogenital infection.^1^ Reactivation of latent infection causes polyomavirus nephropathy (PyVN) in < 10% of kidney allograft recipients, and, rarely, in the native kidney in immunocompromised individuals. BKPyV nephropathy (BKPyVN) is characterized by elevated levels of BKPyV DNA in urine and plasma and shedding of “decoy” cells in the urine, which form the basis of screening programs and presumptive diagnosis. Confirmation requires immunohistochemical staining of the kidney for polyoma antigens (e.g., SV40 large T antigen). BKPyVN is graded histologically based on the polyoma virus load, extent of interstitial fibrosis (Banff ci score)^2^ and interstitial inflammation,^3^ with worse outcomes associated with higher grades. There are no specific therapies for BKPyVN, and management consists of reducing immunosuppressive therapy to restore viral clearance, with poor outcomes associated with ongoing productive viral infection.

Previous studies have reported tubular basement membrane (TBM) deposits of IgG and/or complement in 25% to 50% of BKPyVN.^4^, ^5^, ^6^ This finding has been linked to more severe BKPyVN; however, its biologic and clinical significance are uncertain. Wu *et al.*^7^ provide novel evidence for dynamic complement activation in BKPyVN with TBM immune complex deposits. In a retrospective single center cohort of 154 cases (including 4 native kidneys), C4d staining of TBM was present in 84% of BKPyVN, including 27% with diffuse staining (involving > 50% of TBM). C4d staining was seen around infected and noninfected tubules and correlated with Banff polyoma virus load, i, t, ci, ct scores, and BKPyVN stage (including 94% of Banff class 3 cases). Notably, TBM C4d staining correlated with worse outcomes and was diminished in repeat biopsies associated with clinical improvement. Costaining for IgG and C1 staining was found in 100% and 80% of cases, respectively, thereby supporting classical pathway complement activation, and immune type electron dense deposits were identified in 60%. Importantly, controls without BKPyVN and/or showing extensive tubulointerstitial scarring demonstrated significantly less TBM C4d staining and no IgG staining, supporting their specificity for BKPyVN. These interesting findings demonstrate that complement activation is not uncommon in BKPyVN and may have pathogenic and prognostic implications.

The pathogenesis of BKPyVN involves virus replication causing tubular epithelial damage and humoral and cellular immune responses leading to intrarenal inflammation and/or fibrosis. Importantly, the extent of interstitial fibrosis appears to be the main pathologic determinant of kidney survival in BKPyVN, emphasizing the need for early detection and prompt suppression of viral replication. The findings of Wu *et al.* indicate that TBM immune-complex deposition and complement activation may contribute to the severity of BKPyVN.^7^ Whether complement activation is the cause or consequence of severe tissue injury remains unknown and has implications for management, because therapies that reduce antibody formation or block complement activation are unlikely to be beneficial in diffusely staining cases with advanced fibrosis. Clearly, any proposal to increase immunosuppression in BKPyVN will first require convincing evidence of pathogenicity and potential reversibility.

The origin and nature of TBM immune deposits in BKPyVN are unknown; however, their occurrence in native kidneys as well as allografts argues against a sole role for alloimmunity. The absence of glomerular deposits argues against (but does not exclude) deposition of circulating immune complexes and suggests *in situ* TBM immune complex formation. The inflammatory cell infiltrates in BKPyVN reflect cell-mediated immunity and it is conceivable that polyoma virus antigens also trigger a humoral response, leading to immune complex deposition at sites of polyoma virus–induced tubular injury, perhaps in BKV genotype mismatched donor-recipient pairs. It is also possible that severe cellular injury in high grade PyVN unmasks epithelial allo/autoantigens that induce immune complex deposition in the adjacent TBM. By analogy, autoantibodies to megalin produce similar findings of TBM and Bowman’s capsule immune-complex and complement deposits.^8^ It would also be interesting to examine the IgG heavy chain subtype(s) in PyVN with TBM deposits, given the pathologic similarities to IgG4-related interstitial nephritis.

What are the diagnostic implications of these findings? Most pathology laboratories already stain renal allograft biopsies for C4d, to detect peritubular capillary staining in antibody mediated rejection, and this staining pattern is readily distinguished from the granular TBM staining occurring in PyVN (Figure 1). Thus, the detection of TBM C4d staining (and immunoglobulin) in a kidney allograft biopsy, particularly if diffuse, should prompt consideration of PyVN and correlation with plasma (or urine) BKPyV DNA levels and SV40 immunostaining. Of note, Wu *et al.* used immunohistochemistry for C4d on archival formalin-fixed tissue and describe increased sensitivity and interpretability compared with immunofluorescence microscopy on frozen or paraffin-embedded tissue.^7^ However, many transplant centers perform immunofluorescence microscopy staining for C4d on frozen tissue, and a side-by-side comparison with immunohistochemistry staining is necessary before adopting immunohistochemistry as the optimal modality for diagnosing BKPyVN with TBM C4d staining.

**Figure 1:**
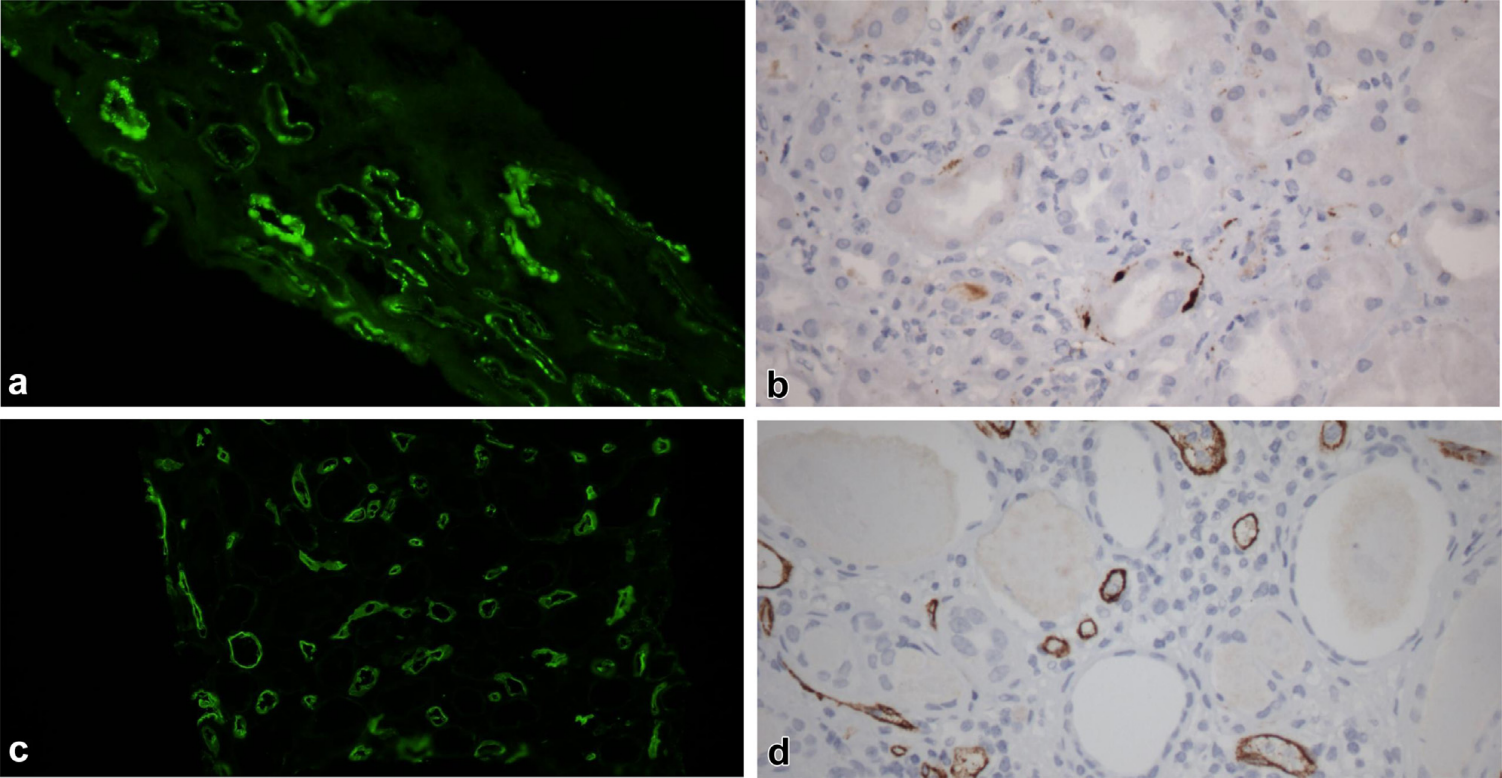
(a and b) C4d staining of tubular basement membranes in BK polyoma virus nephropathy. (c and d) C4d staining of peritubular capillaries in antibody-mediated rejection ([a and c], immunofluorescence staining of frozen tissue; [b and d] immunohistochemical staining of formalin fixed paraffin embedded tissue. All original magnifications 60 ×).

In summary, Wu *et al.* provide convincing evidence for complement activation in severe BKPyVN, adding an additional chapter to the saga of BKPyVN. If other studies can replicate these findings, it would support the diagnostic utility of C4d immunohistochemistry staining in BKPyVN. However, a pathogenic role for complement activation is still unproven and would be strengthened if the target antigen(s) could be identified (e.g., by immunoprecipitation and mass spectrometry), along with circulating antibodies that correlate with disease activity. It would also be important to study BKPyV-specific antibodies and cell-mediated immunity in these cases, to determine if complement activation is occurring systemically or is confined to the kidney. Given the relative rarity of this condition, further collaborative studies are necessary before the story of complement activation in BKPyVN is complete.

## Disclosure

The author declared no competing interests.

## References

[1] Kotton C.N., Kamar N., Wojciechowski D. (2024). The second international consensus guidelines on the management of BK polyomavirus in kidney transplantation. Transplantation.

[2] Nickeleit V., Singh H.K., Randhawa P. (2018). The Banff working group classification of definitive polyomavirus nephropathy: morphologic definitions and clinical correlations. J Am Soc Nephrol.

[3] Hirsch H.H., Randhawa P.S., AST Infectious Diseases Community of Practice (2019). BK polyomavirus in solid organ transplantation-guidelines from the American society of transplantation infectious diseases community of practice. Clin Transpl.

[4] Bracamonte E., Leca N., Smith K.D. (2007). Tubular basement membrane immune deposits in association with BK polyomavirus nephropathy. Am J Transplant.

[5] Hever A., Nast C.C. (2008). Polyoma virus nephropathy with simian virus 40 antigen-containing tubular basement membrane immune complex deposition. Hum Pathol.

[6] Batal I., Zainah H., Stockhausen S. (2009). The significance of renal C4d staining in patients with BK viruria, viremia, and nephropathy. Mod Pathol.

[7] Wu T., Gou F., Yang S. (2025). A novel perspective on tubular C4d positivity in BK polyomavirus nephropathy. Kidney Int Rep.

[8] Larsen C.P., Trivin-Avillach C., Coles P. (2018). LDL receptor-related Protein 2 (megalin) as a target antigen in human kidney anti-brush border antibody disease. J Am Soc Nephrol.

